# Use of an mHealth Ketogenic Diet App Intervention and User Behaviors Associated With Weight Loss in Adults With Overweight or Obesity: Secondary Analysis of a Randomized Clinical Trial

**DOI:** 10.2196/33940

**Published:** 2022-03-14

**Authors:** Kaja Falkenhain, Sean R Locke, Dylan A Lowe, Terry Lee, Joel Singer, Ethan J Weiss, Jonathan P Little

**Affiliations:** 1 School of Health and Exercise Sciences The University of British Columbia Okanagan Kelowna, BC Canada; 2 Department of Kinesiology Brock University St. Catherines, ON Canada; 3 Eureka Research Platform San Francisco, CA United States; 4 Centre for Health Evaluation and Outcome Sciences School of Population and Public Health The University of British Columbia Vancouver, BC Canada; 5 Cardiovascular Research Institute University of California San Francisco San Francisco, CA United States

**Keywords:** acetone, biofeedback, psychology, diet, ketogenic, mobile apps, overweight, technology, telemedicine, weight loss, mobile phone

## Abstract

**Background:**

Low-carbohydrate ketogenic diets are a viable method to lose weight that have regained popularity in recent years. Technology in the form of mobile health (mHealth) apps allows for scalable and remote delivery of such dietary interventions and are increasingly being used by the general population without direct medical supervision. However, it is currently unknown which factors related to app use and user behavior are associated with successful weight loss.

**Objective:**

First, to describe and characterize user behavior, we aim to examine characteristics and user behaviors over time of participants who were enrolled in a remotely delivered clinical weight loss trial that tested an mHealth ketogenic diet app paired with a breath acetone biofeedback device. Second, to identify variables of importance to weight loss at 12 weeks that may offer insight for future development of dietary mHealth interventions, we aim to explore which app- and adherence-related user behaviors characterized successful weight loss.

**Methods:**

We analyzed app use and self-reported questionnaire data from 75 adults with overweight or obesity who participated in the intervention arm of a previous weight loss study. We examined data patterns over time through linear mixed models and performed correlation, linear regression, and causal mediation analyses to characterize diet-, weight-, and app-related user behavior associated with weight loss.

**Results:**

In the context of a low-carbohydrate ketogenic diet intervention delivered remotely through an mHealth app paired with a breath acetone biofeedback device, self-reported dietary adherence seemed to be the most important factor to predict weight loss (*β*=–.31; t_54_=–2.366; *P*=.02). Furthermore, self-reported adherence mediated the relationship between greater app engagement (from c=–0.008, 95% CI –0.014 to –0.0019 to c’=–0.0035, 95% CI –0.0094 to 0.0024) or higher breath acetone levels (from c=–1.34, 95% CI –2.28 to –0.40 to c’=–0.40, 95% CI –1.42 to 0.62) and greater weight loss, explaining a total of 27.8% and 28.8% of the variance in weight loss, respectively. User behavior (compliance with weight measurements and app engagement) and adherence-related aspects (breath acetone values and self-reported dietary adherence) over time differed between individuals who achieved a clinically significant weight loss of >5% and those who did not.

**Conclusions:**

Our in-depth examination of app- and adherence-related user behaviors offers insight into factors associated with successful weight loss in the context of mHealth interventions. In particular, our finding that self-reported dietary adherence was the most important metric predicting weight loss may aid in the development of future mHealth dietary interventions.

**Trial Registration:**

ClinicalTrials.gov NCT04165707; https://clinicaltrials.gov/ct2/show/NCT04165707

**International Registered Report Identifier (IRRID):**

RR2-10.2196/19053

## Introduction

### Background

Overweight and obesity are strongly associated with a number of chronic health conditions, including cardiovascular disease and type 2 diabetes [1]. Approximately 39% and 13% of adults worldwide are living with overweight and obesity, respectively [2], and it is estimated that 40% of adults living in the United States are attempting a weight loss diet every year [3]. Although various diets can be used to achieve a reduction in body weight, successful weight loss requires a sustained decrease in caloric intake that necessitates behavior modification for long-term dietary adherence. For example, self-monitoring of dietary intake and body weight has been shown to aid in achieving weight loss [4]. Mobile technology, such as smartphone mobile health (mHealth) apps, now offers a platform for innovative and highly scalable interventions to be delivered to a broad audience. Apps can offer dietary guidance and assist in implementing behavior changes; for example, through interaction-enabled (eg, tracking) features, notification reminders, and educational content.

A low-carbohydrate, high-fat ketogenic diet is a popular weight loss diet that has robust impacts on metabolism [5]. A ketogenic diet aims to restrict carbohydrate intake to enable the liver to produce ketone bodies (acetoacetate, acetone, and beta-hydroxybutyrate) from free fatty acids, which can be used as an alternative fuel source and which give the diet its name. Although such diets have been shown to be a viable method to successfully lose weight [6], they require knowledge on the part of the dieter related to the macronutrient composition of foods to restrict carbohydrate intake appropriately to achieve ketosis. The diet’s complex nature may present a burden of entry to people trying to lose weight by means of a ketogenic diet because it can be difficult for people wishing to lose weight to know which foods to eat to maintain a state of ketosis.

### *Keyto* App

*Keyto* Inc (*Keyto*) is a company offering an app that provides a Mediterranean-based ketogenic diet intervention. With in-app resources such as recipes, meal plans, and informative articles, the program guides users to follow a low-carbohydrate diet with a focus on fats that fit the Mediterranean guidelines [7] to promote ketosis while counteracting detrimental blood lipid changes known to commonly arise with ketogenic diets high in saturated fat [8]. The app is paired with a hand-held device that measures acetone levels in the breath and serves as a noninvasive biofeedback measure of ketosis, while allowing for self-monitoring of dietary compliance and providing insight into how different foods affect fat burning and ketosis. In a previous randomized clinical trial, the app was shown to be effective for inducing weight loss and improving cardiometabolic health (ie, markers of glycemic control and liver damage) without worsening blood lipid profiles [9]. However, it is currently unknown how adults who are seeking to lose weight engage with the app and what might predict success of such a weight loss intervention. The purpose of this study is therefore to examine user behavior of adults using the *Keyto* weight loss app in a real-world setting and to identify intervention-specific (ie, app use– and adherence-related) behaviors that would predict successful weight loss.

## Methods

### Design

This study is a secondary analysis of app use and outcome data from the intervention group of a previous mHealth-based randomized clinical trial [9]. The original trial examined weight loss and cardiometabolic risk between participants receiving the *Keyto* app paired with a breath acetone biofeedback device and those receiving WW International Inc’s WW app (formerly Weight Watchers International Inc is now WW International Inc) as an active comparator group over the course of 12 (primary end point) and 24 (secondary end point) weeks, as per registration on ClinicalTrials.gov (NCT04165707) and the published protocol (DERR1-10.2196/19053) [10]. The trial was conducted remotely out of Canada with participating individuals living in California. This paper aims to (1) examine characteristics and behaviors of users in this trial as they relate to dietary adherence and app use and (2) identify predictors of weight loss.

### Ethics Approval

This study was approved by the University of British Columbia’s clinical research ethics board (H19-01341) and all participants provided written informed consent digitally prior to data collection.

### Participant Recruitment and Study Flow

As described previously [9,10], participants were recruited through web-based advertisements and an email listserv. Interested participants completed a web-based questionnaire to determine eligibility and, if deemed eligible, scheduled a phone call with a research team member to confirm eligibility and clarify any remaining questions. After the phone call, participants provided informed consent and, if randomized to the *Keyto* intervention, downloaded the app using a study-provided username and password to access the intervention and ensure anonymity. In addition, participants were sent a Bluetooth scale (iHealth Lina) that transmitted weight data to a cloud-based server to be accessed by the research team. The primary intervention phase was 12 weeks, with a secondary end point at 24 weeks, during which participants were asked to follow the dietary intervention based on guidance through the app.

### Mobile Weight Loss Intervention

The mobile intervention program was delivered entirely remotely through the app and without in-person interaction with the research team. The app provided to the intervention group was developed by a multidisciplinary team, including a cardiologist, an engineer, and a physician, and is commercially available. Details of the intervention have been previously described [9,10], but briefly, users are encouraged to follow a low-carbohydrate ketogenic diet with a focus on consuming fats from plant- (eg, olive oil and avocado) and fish-based (eg, salmon) sources that fit the Mediterranean diet pattern [7]. Users are not required to track their food intake but are encouraged to stick to recommended portion sizes and eat until satiety. In-app articles provide meal ideas, suggested shopping lists, advice to avoid common pitfalls, and background information about the ketogenic diet. Users can also join support groups to engage with other users.

An accompanying biofeedback device that pairs with the app measures acetone in the breath as a biomarker of ketosis [11]. The pen-sized device contains a nanostructured gas sensor with a semiconducting metal oxide core selective to acetone and each sensor is individually stabilized and calibrated during the production process. Upon breathing into the device, users receive a *Keyto* Level ranging from 0 (lowest) to 6+ (highest) as an indicator of the degree of ketosis and as a surrogate for fat loss [12]. In case of a lower score (0-3), participants are instructed to further restrict carbohydrate intake and prioritize high-fat foods, whereas in case of a higher score (≥4), participants are encouraged to continue with their current dietary habits. Participants in this trial were asked to use the accompanying breath acetone biofeedback device 3 times daily (first thing in the morning and before lunch and dinner).

### Measures

#### Weight Loss Data

Participants were asked to weigh themselves daily on the study-provided Bluetooth scale. As described previously [9,10], baseline weight was considered the first weight measurement on the start day of the trial or, if no weight was recorded on that date, the weight measurement closest to 8 AM of the start date. The follow-up weight measurement was calculated as the average (mean) weight recorded across the final (ie, 12th and 24th) week of the intervention period to minimize the influence of daily weight fluctuations. Change in body weight and percentage of baseline body weight lost were calculated daily, at the primary end point after 12 weeks, and at the secondary end point after 24 weeks.

#### Adherence

Adherence to the intervention was quantified through the following metrics: (1) compliance with daily weight measurements expressed as the number of days with a weight measurement divided by the total number of days across the intervention period and (2) self-reported dietary adherence assessed weekly through a web-based survey as the response to the question: “To what extent were you able to stick to your diet in the past week?” on a 5-point Likert scale ranging from 0 (not at all) to 4 (completely). Anonymized scale data and questionnaire responses were exported at the end of the trial and summed at weekly intervals to generate adherence metrics for each participant across the intervention period.

#### Engagement With Dietary Intervention

Further to the aforementioned adherence metrics, engagement with the dietary intervention was assessed through (1) *Keyto* Levels obtained through use of the breath acetone biofeedback device and (2) the number of engagements with the *Keyto* app.

#### Questionnaires

Participants were sent a baseline questionnaire upon enrollment in the trial and weekly and monthly questionnaires throughout the intervention period. The baseline questionnaire assessed socioeconomic demographics. Weekly questionnaires assessed self-reported adherence and asked about cravings, mood, and energy (Multimedia Appendix 1). These questionnaires were designed for the purpose of this study by the research team in collaboration with the *Keyto* medical director to provide simple measures of manipulation fidelity and self-reported dietary adherence. Monthly questionnaires collected dietary intake data through the Automated Self-Administered 24-Hour Dietary Recall tool [13]. At the end of the primary intervention phase at 12 weeks, participants were asked about the impact of the COVID-19 pandemic on their dietary habits. This questionnaire was self-designed because at the time of study development, COVID-19 had not been anticipated.

### Statistical Analysis

All statistical analyses were performed using R software (version 3.6.2; R Foundation for Statistical Computing), except for the mediation analysis, which was performed using SPSS software (version 25.0; IBM Corp).

Our first aim is to describe characteristics and adherence- and app use–related behaviors of users in this trial across the entire primary (12 weeks) and secondary (24 weeks) intervention periods. Baseline characteristics and questionnaire responses were summarized as mean (SD) for continuous data and n (%) for categorical data, unless otherwise stated. To investigate time-based data patterns of weight loss, participants were divided into 3 groups according to percentage baseline weight lost at 12 weeks (ie, <5%, >5% to <10%, and >10%).

Our second aim is to identify predictors of weight loss. Because of the complexity of the involved analyses, the greater data availability for the primary 12-week intervention period, and the primary outcome of the trial being weight loss at 12 weeks, we focused our investigation on the predictors of weight loss across 12 weeks. To explore data patterns over time of the adherence metrics, participants were divided into 2 groups according to whether or not clinically significant weight loss (defined as >5% of baseline weight) [14] was achieved at 12 weeks. Z-scores for each predictor variable over time were calculated as the mean of the sample subtracted from the observed value divided by the SD of the sample for that variable at each time point. A linear mixed model was used to evaluate the effect of successful group membership (ie, whether or not the participant achieved >5% weight loss) and the interaction of the effect over time on adherence metrics; the model included time (week and month), success (>5% weight loss or <5% weight loss), and the interaction of time and success as fixed factors and participant as a random factor. Effect estimates (ie, estimated difference in z-scores) for simple effects of success group (in the case of a significant effect of success) or differences in slopes of time for success averaged over the entire intervention period (in the case of a significant interaction effect) with a 95% CI were calculated.

Potential predictor variables (ie, variables of potential relevance in predicting weight loss) related to app use and dietary adherence were explored in a pairwise Spearman rank correlation matrix and included in a multiple linear regression model. Group means of weight loss were compared across tertiles of significant predictor variables of interest using a 1-way analysis of variance. All assumptions were met, and a post hoc Tukey Honestly Significant Difference test was conducted to correct for multiple comparisons.

Similarly, potential predictors of weight loss were further explored through a mediation analysis between app-specific features (ie, average *Keyto* Level and total number of app engagements across the 12-week intervention period) and weight loss to explore causal mechanisms and investigate potential explanatory pathways. The PROCESS macro (version 3.3) for SPSS was used to estimate the hypothesized mediating effects of weight loss using direct and indirect effects based on 5000 bootstrapping samples at 95% bias-corrected CIs [15]; CIs that do not cross zero (either all positive or negative) suggest that the true effect is not zero and that the null hypothesis can be rejected. 

## Results

### Participants

A total of 77 participants were randomized to the ketogenic diet mobile app group within the larger remote randomized clinical trial, as previously described [9]. Of the 77 participants, 1 (1%) was deemed ineligible after enrollment and 1 (1%) did not start; during the primary 12-week intervention phase, 3 (4%) discontinued the intervention, 10 (13%) were lost to follow-up, and 2 (3%) did not log a follow-up weight. The remaining participants (n=60) were included in weight-related analyses for the primary intervention phase, and partial data from all participants was used for any other analyses as available. At the secondary 24-week end point, weight data from 55% (42/77) of the participants were available. Available data from all participants were included in analyses related to adherence and user behavior. Self-reported baseline characteristics of participants assessed through a questionnaire before the intervention are presented in Table 1.

**Table 1:** Baseline characteristics of participants (N=75).

Characteristics	Total	Female	Male	Nonbinary
Participants, n (%)	75 (100)	53 (71)	20 (27)	2 (2)
Age (years), mean (SD)	42 (11)	41 (10)	43 (13)	33 (9)
Weight (kg), mean (SD)	94.7 (17.1)	90.7 (16.7)	104.2 (14.3)	106.1 (18.8)
BMI, mean (SD)	33.5 (4.7)	33.5 (4.9)	33.1 (4.5)	34.4 (4.7)

### Change in Body Weight

Mean weight loss at 12 weeks was –5.6 (SD 4.5) kg, which equated to –5.8% (SD 4.5%) of initial body weight. Of the participants logging a follow-up weight, 53% (32/60) lost >5% and 18% (11/60) lost >10% of baseline body weight (Table 2).

**Table 2:** Weight loss outcomes.

Outcome measure		Total		Female	Male	Nonbinary
**Primary intervention end point at 12 weeks (N=60)**						
	Participants, n (%)	60 (100)		42 (70)	16 (27)	2 (3)
	Change in body weight (kg), mean (SD)	–5.6 (4.5)		–5.1 (4.1)	–6.9 (5.3)	–7.1 (3.0)
	Change in body weight (%BBW^a^), mean (SD)	–5.8 (4.5)		–5.5 (4.7)	–6.5 (4.4)	–6.5 (1.6)
	Change in BMI, mean (SD)	–1.9 (1.5)		–1.9 (1.5)	–2.1 (1.6)	–2.3 (0.9)
	Lost >5% initial body weight, n (%)	32 (53)		21 (50)	9 (56)	2 (100)
	Lost >10% initial body weight, n (%)	11 (18)		6 (14)	5 (31)	0 (0)
**Secondary intervention end point at 24 weeks (N=42)**						
	Participants, n (%)		42 (100)	30 (71)	11 (26)	1 (3)
	Change in body weight (kg), mean (SD)		–8.5 (6.4)	–8.3 (6.4)	–9.1 (7.1)	–9.6 (—^b^)
	Change in body weight (%BBW), mean (SD)		–8.7 (6.9)	–8.9 (7.5)	–8.4 (5.6)	–8.1 (—)
	Change in BMI, mean (SD)		–3.0 (2.3)	–3.0 (2.5)	–2.7 (2.0)	–3.0 (—)
	Lost >5% initial body weight, n (%)		24 (57)	18 (60)	5 (45)	1 (100)
	Lost >10% initial body weight, n (%)		15 (36)	11 (37)	4 (36)	0 (0)

^a^%BBW: percentage of baseline body weight.

^b^Not available.

**Figure 1:**
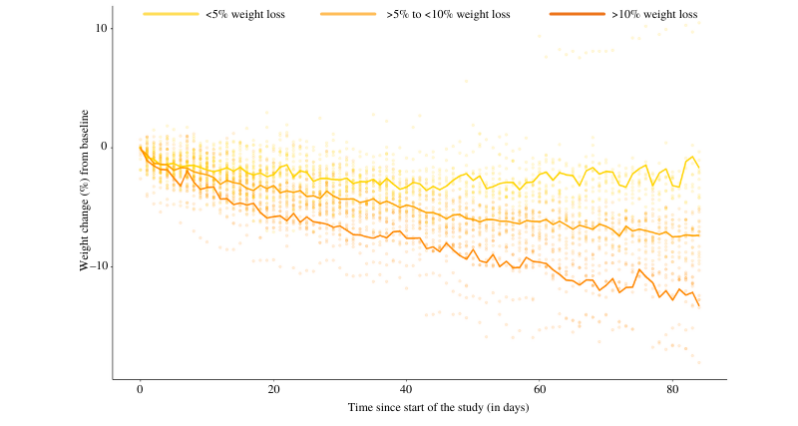
Individual change in body weight (calculated as daily percentage change from baseline based on measurements recorded from an at-home Bluetooth scale) are shown for each participant over time throughout the duration of the study. Daily mean values over time for each group based on end-of-study weight loss at 12 weeks are represented in solid lines (dark orange, >10% weight loss; light orange, >5% to <10% weight loss; and yellow, <5% weight loss).

Daily change in body weight (expressed as a percentage of baseline body weight) across the 12 weeks separated by weight loss groups (ie, weight loss of <5%, >5% to <10%, and >10%) is shown in Figure 1. Body weight decreased over time in all groups; however, the slope of the decrease was steepest (ie, the change was greatest) in the group of participants who lost >10% of their initial body weight and the magnitude of change was smallest in the group of participants who lost <5% of their starting weight, suggesting overall consistency of weight loss success across the intervention period. Mean weight loss at the secondary 24-week time point was –8.5 (SD 6.4) kg or –8.7% (SD 6.9%) of initial body weight, indicating durability of the observed decrease in body weight (Table 2).

### Aim 1: User Behavior and Adherence Over Time

**Figure 2:**
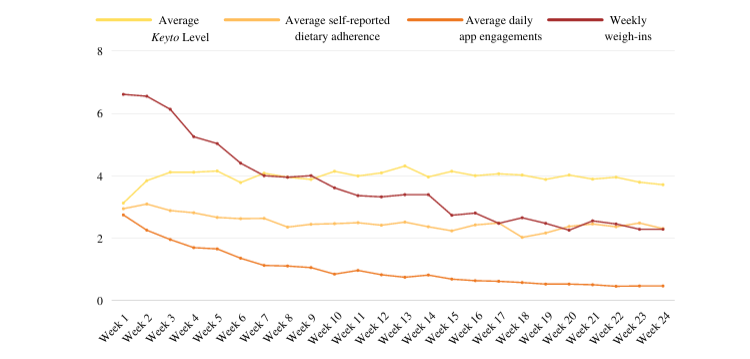
App use metrics (yellow, average *Keyto* Level obtained through breath acetone biofeedback device; dark orange, average daily number of engagements with the *Keyto* app), self-reported dietary adherence (light orange), and average number of weekly weight measurements (brown) averaged across all participants throughout the intervention. Mean values are shown.

Participants were instructed to use the app and accompanying biofeedback device as dietary guidance throughout the intervention and to report dietary adherence on a weekly basis through a web-based questionnaire; self-reported adherence across 12 weeks was moderately high, with an average score of 2.6 (SD 0.2) on a 5-point Likert scale. The average number of engagements with the app across the entire primary intervention period was 123 (SD 91), or 1.5 (SD 0.6) engagements daily, and the average *Keyto* Level was 3.9 (SD 0.3). On average, participants weighed themselves 4.7 (SD 1.2) times per week. Across both the primary and secondary intervention phases, the average self-reported adherence was 2.5 (SD 0.3), *Keyto* Levels were 4.0 (SD 0.2), number of weekly weight measurements was 3.7 (SD 1.4), and the number of engagements with the app was 171 (SD 155), or approximately once per day (Figure 2).

**Figure 3:**
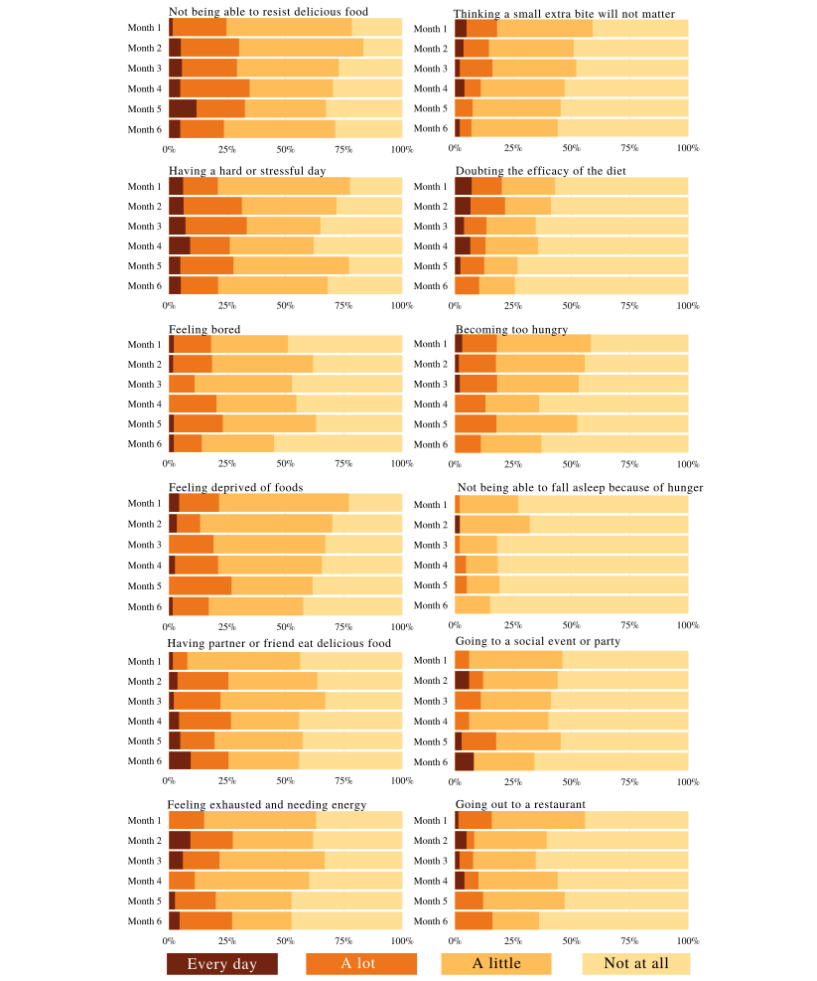
Aggregated responses to questionnaires sent through email, asking participants, “How does the following affect your ability to stick to your diet?” on a 4-point Likert scale, ranging from “Not at all” to “Every day.”.

To further assess how different factors might affect self-reported dietary adherence, we asked participants how strongly a given situation would affect their ability to stick to their diet on a scale of 1 (not at all) to 4 (every day). Descriptive data from these questionnaires are presented in Figure 3.

### Aim 2: Predictors of Weight Loss at 12 Weeks

#### Correlation of Potential Predictor Variables

**Figure 4:**
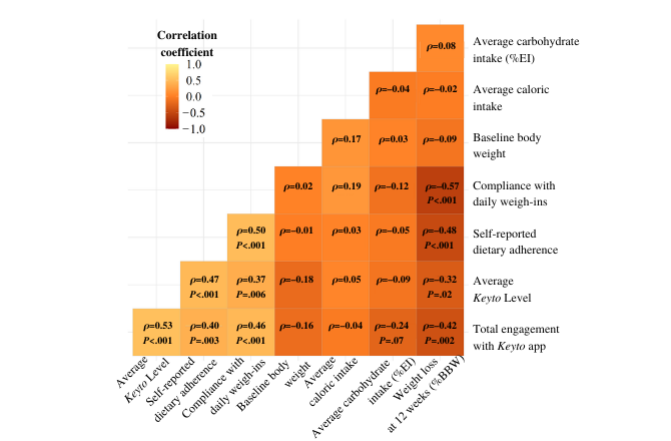
Pairwise Spearman rank correlation matrix of adherence-, app use–, and diet-related variables. %BBW: percentage of baseline body weight; %EI: percentage of energy intake.

A correlation matrix of the assessed app use–, adherence-, and diet-related variables averaged across the primary 12-week intervention period (average *Keyto* Level, app engagement, self-reported caloric intake, carbohydrate intake as percentage of daily energy intake, compliance with daily weigh-ins, and self-reported dietary adherence), baseline body weight, and weight loss as a percentage of baseline body weight lost is shown in Figure 4. The strongest negative correlations were observed between weight loss and compliance with daily weight measurements (*ρ*=–0.57; *P*<.001), self-reported dietary adherence (*ρ*=–0.48; *P*<.001), and app engagement (*ρ*=–0.42; *P*=.001), suggesting that participants who weighed themselves more regularly, reported greater dietary adherence, and engaged more often with the app also achieved greater weight loss. In addition, average *Keyto* Levels were significantly correlated with weight loss (*ρ*=–0.32; *P*=.02), indicating greater weight loss with higher *Keyto* Levels. The strongest positive correlations were observed between average *Keyto* Levels and engagement with the *Keyto* app (*ρ*=0.53; *P*<.001), suggesting that participants who engaged more with the app also achieved higher *Keyto* Levels, and between self-reported dietary adherence and compliance with daily weight measurements (*ρ*=0.50; *P*<.001), indicating high agreement between these 2 adherence measures.

#### Time-Based Data Patterns

**Figure 5:**
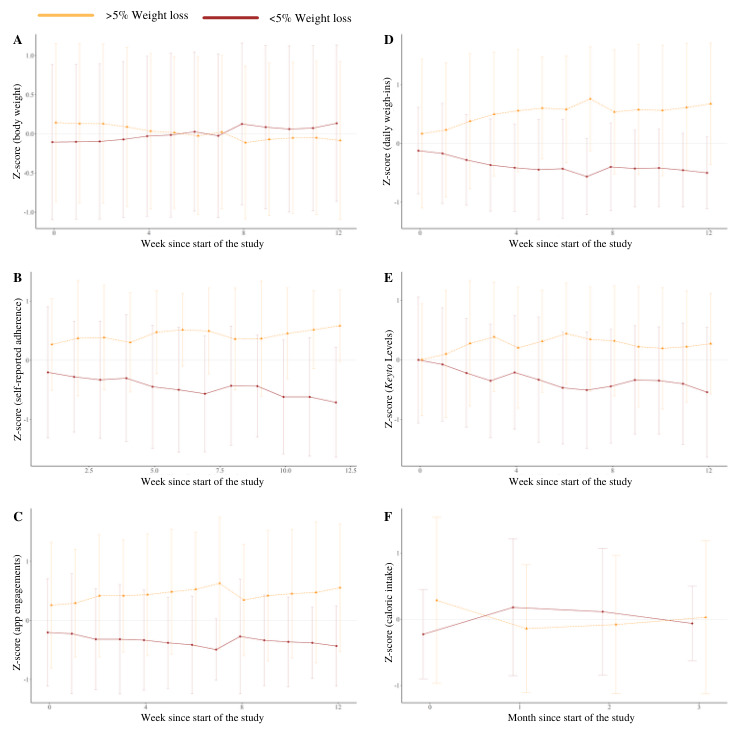
Average z-scores (the mean of the sample subtracted from the observed value divided by the SD of the sample at each time point) comparing time-series patterns of variable means between participants who lost >5% of initial body weight (light orange) and those who lost <5% of baseline body weight (dark orange). Mean values (bold lines) and SD (shaded lines) are shown for (A) body weight, (B) self-reported dietary adherence, (C) app engagement, (D) compliance with daily weight measurements, (E) average *Keyto* Levels, and (F) self-reported caloric intake.

To investigate more detailed patterns of assessed variables across time (as opposed to values averaged over the entire intervention period), we plotted the average z-scores (a measure of deviation from the average value at each time point) of adherence-, diet-, and app use–related variable means between the group of participants who achieved clinically significant weight loss of >5% initial body weight (termed as *successful* participants) and the group of participants who did not (ie, participants who lost <5% initial body weight). Variable trends are shown in Figure 5. As expected, there was a significant interaction between success and time for body weight (Figure 5A), with the slope for the successful group decreasing significantly more over time (–0.03, 95% CI –0.034 to –0.027; *P*<.001). Likewise, there was a significant interaction for self-reported dietary adherence (Figure 5B), app engagement (Figure 5C), and compliance with daily weight measurements (Figure 5D): self-reported dietary adherence (0.07, 95% CI 0.04-0.10; *P*<.001), engagement with the *Keyto* app (0.03, 95% CI 0.01-0.05; *P*=.005), and compliance with daily weight measurements (0.06, 95% CI 0.03-0.08; *P*<.001) were higher over time in the successful group of participants. Furthermore, there was a significant effect of success group for these variables: successful participants reported higher dietary adherence (0.47, 95% CI 0.13-0.81; *P*=.007), engaged with the app more often (0.62, 95% CI 0.28-0.97; *P*<.001), and weighed themselves more consistently (0.55, 95% CI 0.25-0.86; *P*<.001). A significant interaction effect was detected for average *Keyto* Levels (Figure 5E): participants in the successful group had higher *Keyto* Levels over time (0.08, 95% CI 0.05-0.10; *P*<.001). No significant interaction or main effect was observed for caloric intake (Figure 5F).

#### Regression Model

Following up on the aforementioned correlative analyses, we next sought to identify what process variables might be associated with (or be predictive of) weight change; to this end, we built a regression model that included the adherence- and app use–related variables, namely (1) compliance with weight measurements, (2) average self-reported dietary adherence, (3) total number of app engagements, (4) average *Keyto* Levels, and (5) baseline body weight (Table 3).

**Table 3:** Regression model identifying potential predictor variables of weight loss.

Predictor variable	Values, *β* (SE)^a^	*t* test (*df*)	*P* value
Compliance with daily weight measurements	–.20 (0.13)	–1.580 (54)	.12
Self-reported dietary adherence	–.31 (0.13)	–2.366 (54)	.02
Total number of app engagements	–.14 (0.13)	–1.069 (54)	.29
Average *Keyto* levels	–.06 (0.13)	–0.469 (54)	.64
Baseline body weight	–.36 (0.11)	–3.420 (54)	.001

^a^Standardized coefficient.

The model (adjusted *R^2^*=0.40; *F*_5,54_=8.937; *P*<.001) identified self-reported dietary adherence (*β*=–.31; t_54_=–2.366; *P*=.02) and baseline body weight (*β*=–.36; t_54_=–3.420; *P*=.001) as significant predictors. Compliance with daily weight measurements (*β*=–.20; t_54_=–1.580; *P*=.12) was near statistical significance, whereas engagement with the *Keyto* app (*β*=–.14; t_54_=–1.069; *P*=.29) and average *Keyto* Levels (*β*=–.06; t_54_=–0.469; *P*=.64) were not identified as statistically significant predictors.

**Figure 6:**
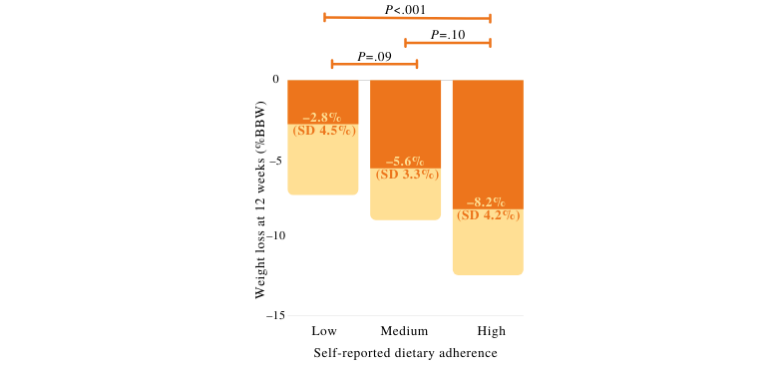
Mean weight loss at the primary intervention end point of 12 weeks of participants in the lowest (left), medium (center), or highest (right) tertile of average self-reported dietary adherence assessed weekly through a questionnaire. One-way analysis of variance with Tukey post hoc tests comparing the differences in group means was conducted. Mean (dark orange) and SD (light orange) are shown. %BBW: percentage of baseline body weight.

To further evaluate the relationship of weight loss with self-reported dietary adherence as a significant predictor variable, we split participants into tertiles based on low *versus* medium *versus* high average self-reported dietary adherence and compared weight loss among these groups (Figure 6). Weight loss was significantly different among the tertiles (*F*_2,57_=8.586; *P*<.001); post hoc testing revealed a statistically significant difference in weight loss between the participants who reported lowest dietary adherence and those who reported highest dietary adherence (*P*<.001).

#### Mediation Analysis

**Figure 7:**
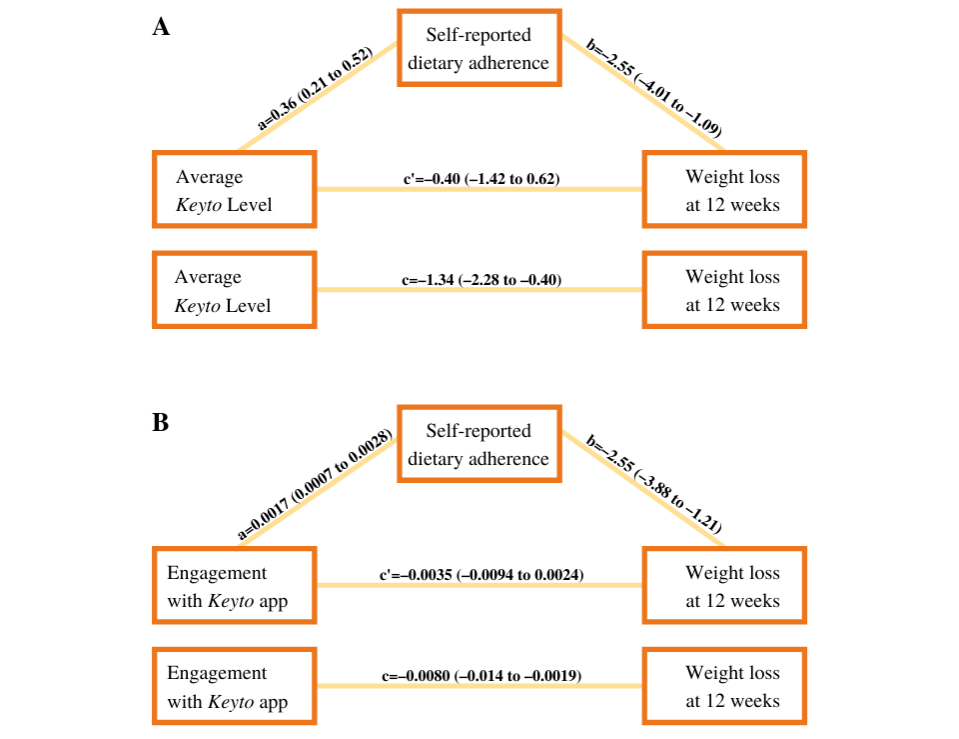
Unstandardized effects with 95% CIs of the direct and indirect mediation effects of self-reported dietary adherence on (A) average *Keyto* Levels and (B) total engagement with the *Keyto* app on body weight loss at the end of the primary intervention phase at 12 weeks.

Finally, because we were interested in the effect of features specific to the *Keyto* app, we set out to further examine explanatory pathways of the relationship between average *Keyto* Levels or overall engagement with the app, weight loss, and the previously identified predictor, that is, self-reported adherence to the dietary intervention. Figure 7 depicts the results of these 2 mediation analyses. Self-reported dietary adherence fully mediated the relationship between average *Keyto* Level and weight loss at 12 weeks (Figure 7A). When self-reported dietary adherence was added to the model, the direct relationship between average *Keyto* Level and 12-week weight loss became nonsignificant (see change from *c* to *c’*). Average *Keyto* Level accounted for 12.3% of the variance in weight loss when included on its own, and 27.8% was explained when dietary adherence was included in the mediation model. Similarly, self-reported dietary adherence fully mediated the relationship between the overall engagement with the *Keyto* app and 12-week weight loss (Figure 7B). When self-reported dietary adherence was added to the model, the direct relationship between app engagement and 12-week weight loss became nonsignificant (see change from *c* to *c’*). On its own, app engagement accounted for 10.7% of the variance in weight loss, whereas 28.8% of the variance was explained when dietary adherence was included in the mediation model.

Higher average *Keyto* Levels and greater engagement with the *Keyto* app were associated with greater ability to adhere to the dietary intervention, which in turn was associated with 12-week weight loss. These findings suggest that using the *Keyto* app more frequently and achieving higher *Keyto* Levels caused individuals to better adhere to the dietary intervention, which resulted in greater weight loss.

### Impact of COVID-19

**Figure 8:**
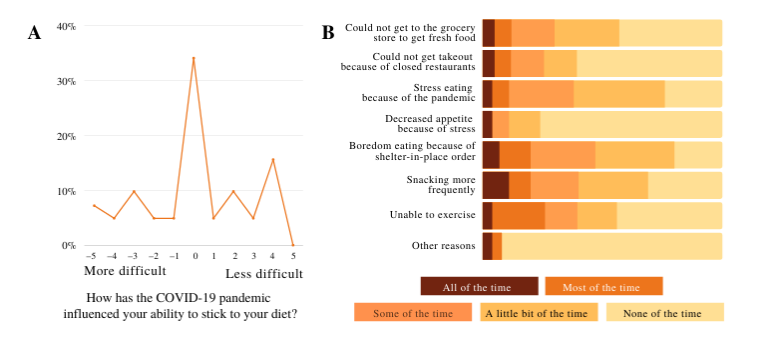
Response to the questionnaires sent through email, asking participants how (A) the COVID-19 pandemic has influenced their ability to stick to their diet on a scale of –5 (more difficult) to +5 (less difficult) and (B) how the listed items affected their ability to stick to their diet with respect to the COVID-19 pandemic on a 5-point Likert scale ranging from “None of the time” to “All of the time.”.

Although the study was designed before the COVID-19 pandemic, most of the components of the trial were conducted during the pandemic. Therefore, at the end of the primary intervention phase, we asked participants to report how the COVID-19 pandemic had influenced their ability to stick to their diet on a scale of –5 (more difficult) to +5 (less difficult). The average response to this question was 0.2 (SD 3.1), suggesting a generally minor impact of the pandemic; however, the large variability indicates that some participants experienced significantly greater challenges because of the pandemic (Figure 8A). Regression analysis revealed that the self-reported impact of COVID-19 on participants’ ability to stick to their diet explained 22.1% of the variance in self-reported dietary adherence (*F*_1,43_=13.45; *P*<.001).

Figure 8B shows aggregated responses to the question of how the listed scenarios affected the participants’ ability to stick to their diet; in particular, stress eating, boredom eating, and snacking more frequently were identified as posing the greatest challenges to sticking to the diet during the pandemic.

## Discussion

### Study Overview

In this secondary analysis of a previous randomized clinical trial [9], we showed that a smartphone-based mHealth app promoting a low-carbohydrate ketogenic diet high in mono- and polyunsaturated fatty acids was effective at reducing body weight over the course of 12 and 24 weeks. Furthermore, we characterized real-world user behavior with the app and the accompanying breath acetone biofeedback device over the course of the intervention period and investigated differences between the participants who achieved clinically significant weight loss and those who did not. Finally, we identified predictor variables of weight loss and showed that self-reported adherence to the dietary intervention seemed to be the most important factor in predicting weight loss success. This suggests that a relatively simple measure of self-reported adherence could be used to help predict who will be successful with an mHealth app-based dietary weight loss intervention, which could allow for greater individualization of future interventions.

### Relevance of Findings

The weight loss observed in the intervention group in this trial compared favorably with other weight loss–promoting mHealth apps [16] and previous weight reduction trials that used a low-carbohydrate ketogenic diet using more traditional and hands-on (ie, nutritional counseling and in-person group meetings) designs [17]. This suggests that an app paired with a biofeedback device that guides users to follow a low-carbohydrate ketogenic diet is a feasible and effective approach for promoting weight loss in a pragmatic real-world setting that is highly scalable and presents low burden of entry.

To our knowledge, this is the first study evaluating user behavior of a smartphone-based weight loss app using a low-carbohydrate ketogenic diet. Because of the nature of the diet, substantial knowledge is required to identify suitable food items that fit this dietary plan. In addition, a ketogenic diet offers the opportunity for self-monitoring of a fat loss biomarker (ie, breath acetone) other than, or in addition to, the weight scale, which is unique to this dietary pattern. The breath acetone biofeedback device used in this study is of particular interest because most available methods of measuring ketone bodies are invasive (eg, measuring beta-hydroxybutyrate through finger pricks) or offer only a rough proxy of ketosis (eg, measuring acetoacetate through urine sticks). Our study therefore adds valuable insight into how users interact with an app promoting such a diet without direct in-person supervision and which behaviors lead to successful weight loss.

Similar to previous studies investigating mobile weight loss interventions [18], we found that the measures of dietary adherence and intervention fidelity (ie, engagement with the app and body weight measurements) decreased over time. However, importantly, we showed that this decrease was less pronounced in participants who achieved clinically significant weight loss (ie, >5% baseline body weight) compared with those who did not. Upon further investigation of data patterns over time, we found that this difference in behaviors between *successful* and *unsuccessful* participants was apparent right from the beginning of the intervention, with successful participants reporting greater levels of dietary adherence, weighing themselves more regularly, and engaging more often with the app. Moving from correlative to more causative associations, our mediation analysis further suggests that self-reported dietary adherence was a mechanism through which engagement with the *Keyto* app and higher *Keyto* Levels affected weight loss during the primary intervention period of 12 weeks. Interestingly, the average *Keyto* Level as a direct measure of ketosis and an indirect measure of dietary adherence did not seem to decrease over time; this would suggest that for participants to maintain a state of ketosis, persistent engagement with the app (which, in contrast, did decrease over time) was not required after an initial familiarization period. However, it is important to note that the metric of average *Keyto* Levels in our study is upwardly biased because of response bias (ie, only participants who actively use their breath acetone device will record a *Keyto* Level) and therefore a measure of people who are at least somewhat engaged with the dietary intervention, whereas engagement with the app is representative of all participants regardless of their engagement with the study (ie, a meaningful zero exists for number of app engagements).

Overall, our data support the importance of adherence to the intervention (more so than absolute carbohydrate restriction or level of ketosis) to achieve meaningful weight loss. Although the different measures used to evaluate adherence showed high correlation, indicating a large degree of concordance among them (ie, participants who weighed themselves more regularly generally engaged with the app more often and so on), more detailed analysis implied that different measures of adherence (ie, daily weight measurements, engagement with the app, and self-reported adherence) were differentially associated with weight loss success, supporting the notion of adherence as a multifaceted construct. In particular, our results suggest that self-reported dietary adherence—a subjective assessment provided by participants through weekly questionnaires—was the most important predictor of successful weight loss; future investigations should therefore aim to determine the extent to which objective adherence to the dietary intervention and self-reported dietary adherence are in agreement and which factors influence self-reported adherence, which may in turn inform the design of future dietary app-based interventions. The finding that a relatively simple self-reported assessment of dietary adherence seemed to be the most powerful predictor of successful weight loss also has practical value that could help inform future trials and knowledge translation for the design of app-based interventions. We speculate that evaluating self-reported adherence could help to determine early on in the trial which participants might need extra support (eg, through SMS text messages, push notifications, and phone calls) to maximize weight loss success.

### Limitations

Importantly, our trial design was unable to separate the effect of individual intervention components (eg, breath acetone device, app engagement, and recording of weight measurements); although we attempted to statistically tease apart variables of predictive power, our study may inspire future research with other trial designs (eg, Multiphase Optimization Strategy–based [19]) to further investigate the independent importance of intervention constituents.

Furthermore, our trial was conducted in a real-world setting. Although this pragmatic trial design was chosen on purpose to evaluate the effect of the app-based intervention in a realistic setting, we were therefore limited, for the most part, to self-reported data entries; in particular, we relied on dietary self-report for measures of caloric intake, which can be biased and provide inaccurate estimates [20], and we asked users to report dietary adherence by means of a weekly questionnaire, which represents a subjective estimate of adherence to the intervention as opposed to an objective assessment. Similarly, body weight was not measured by a trained researcher in a laboratory setting but instead self-administered on an at-home Bluetooth scale by the participants, which could potentially introduce bias.

In addition, our findings on user behavior and its change over time are limited to the variables collected within the framework of this trial. It is likely that other variables, including baseline characteristics and other behaviors related to dieting and self-monitoring that were not assessed in this study, are of importance to the success that an individual sees with a given weight loss program. Similarly, our findings are constrained to the evaluated mHealth app promoting a ketogenic diet and may not be as relevant to other dietary interventions.

Notably, our study was conducted throughout the COVID-19 pandemic. Although most participants reported minor effects of the pandemic on their eating plan, some of our psychological measures may have been affected by it. For example, we assessed how going out to a restaurant or a social event affected participants’ ability to stick to their diet; however, because of the statewide shelter-in-place order during the trial, which affected many participants and prohibited restaurant visits or social gatherings, this measure may not accurately reflect the challenges that adults trying to lose weight may encounter under *normal* conditions. Likewise, the greater emotional burden throughout the pandemic [21] may have altered participants’ responses to questions about stress eating or boredom eating.

Finally, our study investigated user behavior and success predictors primarily over a time period of 12 weeks. This represents the effects of a short-term weight loss intervention; therefore, no conclusions can be drawn about the observed relationships on continued weight loss in the context of longer-term dietary interventions or sustained weight maintenance.

### Conclusions

This study adds to the available literature on the use of mHealth technology in assisting self-guided weight loss attempts and supports the notion that even in the contexts of a low-carbohydrate ketogenic diet and mHealth technology, dietary adherence is of crucial importance to achieve the desired reduction in weight. Therefore, our study may inspire future research into how self-reported dietary adherence can be used or enhanced for long-term weight loss and maintenance and inform the design of low-carbohydrate ketogenic dietary mHealth interventions.

## Appendix

Multimedia Appendix 1 Weekly survey used throughout the trial to assess self-reported adherence and cravings, mood, and energy.

## Abbreviations

mHealth: mobile health
