# Supplementary material for: Use of an mHealth Ketogenic Diet App Intervention and User Behaviors Associated With Weight Loss in Adults With Overweight or Obesity: Secondary Analysis of a Randomized Clinical Trial
Source: JMIR Mhealth Uhealth. 2022 Mar 14;10(3):e33940. doi: 10.2196/33940 (PMC8961343; doi:10.2196/33940)
Supplement: Multimedia Appendix 1 [file mhealth_v10i3e33940_app1.docx]

Multimedia Appendix 1 | Weekly survey used throughout the trial to assess self-reported adherence and cravings, mood, and energy

Weekly Measures

To what extent do you believe you were able to stick to the diet as part of this study in the past week?

- 0 - not at all
- 1 - a little bit
- 2 - somewhat
- 3 - a lot
- 4 - completely

How often did you monitor and track your food intake on average each day in the past week?

- 0 times per day
- 1 time per day
- 2 times per day
- 3 or more times per day

How does the following affect your ability to stick to your diet?

|  | 1 Not at all | 2 A little bit (once per week) | 3 A lot (several days per week) | 4 Every day |
| --- | --- | --- | --- | --- |
| Your partner/friend/child is eating something delicious in front of you, and you can't resist |  |  |  |  |
| You're feeling exhausted and need an energy boost |  |  |  |  |
| You are not convinced that a small handful of nuts or other small bite is a big problem |  |  |  |  |
| Going to a social event or party |  |  |  |  |
| Having a really hard or stressful day |  |  |  |  |
| You become too hungry and can't continue to resist eating |  |  |  |  |
| Going out to a restaurant |  |  |  |  |
| You're feeling deprived of foods |  |  |  |  |
| You're not convinced that sticking with this eating plan is really helping |  |  |  |  |
| Having delicious foods in front of you, and you can't resist |  |  |  |  |
| You feel bored |  |  |  |  |
| You can't fall asleep / stay asleep at night because you are too hungry |  |  |  |  |
